# Paralog-specific TTC30 regulation of Sonic hedgehog signaling

**DOI:** 10.3389/fmolb.2023.1268722

**Published:** 2023-11-23

**Authors:** Felix Hoffmann, Sylvia Bolz, Katrin Junger, Franziska Klose, Isabel F. Stehle, Marius Ueffing, Karsten Boldt, Tina Beyer

**Affiliations:** Institute for Ophthalmic Research, Eberhard Karls University Tübingen, Tübingen, Germany

**Keywords:** cilia, IFT, IFT70, TTC30 paralogs, affinity proteomics, Sonic hedgehog signaling, PKA

## Abstract

The intraflagellar transport (IFT) machinery is essential for cilia assembly, maintenance, and trans-localization of signaling proteins. The IFT machinery consists of two large multiprotein complexes, one of which is the IFT-B. TTC30A and TTC30B are integral components of this complex and were previously shown to have redundant functions in the context of IFT, preventing the disruption of IFT-B and, thus, having a severe ciliogenesis defect upon loss of one paralog. In this study, we re-analyzed the paralog-specific protein complexes and discovered a potential involvement of TTC30A or TTC30B in ciliary signaling. Specifically, we investigated a TTC30A-specific interaction with protein kinase A catalytic subunit α, a negative regulator of Sonic hedgehog (Shh) signaling. Defects in this ciliary signaling pathway are often correlated to synpolydactyly, which, intriguingly, is also linked to a rare TTC30 variant. For an in-depth analysis of this unique interaction and the influence on Shh, TTC30A or B single- and double-knockout hTERT-RPE1 were employed, as well as rescue cells harboring wildtype TTC30 or the corresponding mutation. We could show that mutant TTC30A inhibits the ciliary localization of Smoothened. This observed effect is independent of Patched1 but associated with a distinct phosphorylated PKA substrate accumulation upon treatment with forskolin. This rather prominent phenotype was attenuated in mutant TTC30B. Mass spectrometry analysis of wildtype *versus* mutated TTC30A or TTC30B uncovered differences in protein complex patterns and identified an impaired TTC30A–IFT57 interaction as the possible link leading to synpolydactyly. We could observe no impact on cilia assembly, leading to the hypothesis that a slight decrease in IFT-B binding can be compensated, but mild phenotypes, like synpolydactyly, can be induced by subtle signaling changes. Our systematic approach revealed the paralog-specific influence of TTC30A KO and mutated TTC30A on the activity of PRKACA and the uptake of Smoothened into the cilium, resulting in a downregulation of Shh. This downregulation, combined with interactome alterations, suggests a potential mechanism of how mutant TTC30A is linked to synpolydactyly.

## 1 Introduction

Cilia are highly conserved organelles extending from the cellular surface of nearly all eukaryotic organisms. They can be divided into motile and non-motile subtypes (Wheatley, 1995; Pazour and Witman, 2003; Satir and Christensen, 2007; Berbari et al., 2009; Ishikawa and Marshall, 2011; Wheway et al., 2018). Non-motile (primary or sensory) cilia are comprised of several compartments that are crucial for maintaining ciliary function. The axoneme, a microtubule scaffold, provides the basis for intraflagellar transport (IFT). For cilium assembly, proteins are trafficked from the proximal end of the cilium, the basal body, to the distal ciliary tip. At the tip, the transported cargo is released and integrated into the growing cilium (Lee and Chung, 2015). IFT is a bidirectional transport process that is facilitated by large protein complexes and the support of motor proteins. The movement in an anterograde manner is driven by kinesin-2, and the retrograde transport is driven by dynein-2 (Vashishtha et al., 1996; Pazour et al., 1999; Lechtreck, 2015; Hoffmann et al., 2022). The IFT particles are large multiprotein complexes that can be classified into two subcomplexes, IFT-A and IFT-B (Lechtreck, 2015). They contain 6 (A) and 16 (B) unique proteins (Cole et al., 1998). IFT-B can be further separated into a stable core complex, IFT-B1 (IFT88, −81, −74, −70, −52, −46, −27, −25, and −22), which interacts with a peripheral subcomplex, IFT-B2 (IFT172, −80, −57, −54, −38, and −20) (Boldt et al., 2016; Taschner and Lorentzen, 2016). The IFT-B1 protein IFT70/TTC30 is essential for IFT-B stability. The depletion of DYF-1, the IFT70 ortholog in *Chlamydomonas reinhardtii*, results in a reduction of ciliary length (Fan et al., 2010). The IFT70 ortholog in *Danio rerio*, *fleer* (*flr*), led to mutation to an identical phenotype. Here, shortened cilia were connected to a reduction of polyglutamylated tubulin, which was already shown to influence axoneme stability (Pathak et al., 2007). There are two paralogs present in humans, TTC30A and B, which are essential for IFT-B core complex stability. TTC30A and B have an almost identical nucleotide sequence and, as a result, also have a highly similar protein structure, which is conserved across species (Du et al., 2019; Hoffmann et al., 2022). Each human paralog individually interacts with the IFT-B complex and is able to maintain cilia assembly. However, cilia length is decreased, and tubulin polyglutamylation is reduced upon the expression of one paralog only. Concomitant loss of TTC30A and B results in the absence of ciliogenesis, which emphasizes the relevance of TTC30 in IFT-B-mediated ciliary assembly (Fan et al., 2010; Takei et al., 2018; Hoffmann et al., 2022).

The ciliary membrane consists of a particular subset of proteins, such as transmembrane receptors and ion channels. This specific composition, together with the ciliary tip, allows involvement in several signaling pathways (Satir and Christensen, 2007; Rohatgi and Snell, 2010; Hildebrandt et al., 2011; Lee and Chung, 2015; Wheway et al., 2018). So far, Wnt, Notch, Hippo, GPCR, TGF-β, and Sonic hedgehog (Shh) pathways have been linked to the cilium, with Shh being one of the most intensively studied primary cilia-dependent signaling pathways. External stimuli initiate intraciliary interaction cascades. The signal is transduced and regulated, and it subsequently alters the activity of transcription factors, which then translocate to the nucleus and ultimately regulate proliferation, cellular growth, differentiation, and ciliogenesis (Berbari et al., 2009; Mitchison and Valente, 2017; Wheway et al., 2018; Anvarian et al., 2019). Disrupted signaling pathways are often connected to severe diseases (e.g., cancer) or are even lethal in embryonic development, whereas dysregulation leads to rather mild phenotypes. For instance, impaired Shh is connected to synpolydactyly (Towers et al., 2008; Zhu and Mackem, 2017; Zhu et al., 2022). Intriguingly, a previous study discovered a rare missense mutation in TTC30B in a Chinese pedigree. The authors could link the A375V missense mutation to Shh signaling (Du et al., 2019).

In the absence of Hedgehog (Hh) ligand, the 12-pass transmembrane receptor Patched1 (Ptch1) is located at the ciliary membrane and prevents the 7-pass transmembrane protein Smoothened (Smo) from localizing to the cilium (Rohatgi et al., 2007; Rohatgi and Snell, 2010; Arensdorf et al., 2016; Wheway et al., 2018). Additionally, an active G protein-coupled receptor, GPR161, increases intraciliary cAMP levels (Humke et al., 2010). The binding of cAMP to the two regulatory subunits of protein kinase A (PKA) leads to a dissociation of this tetrameric holoenzyme and relieves the inhibition of the two catalytic subunits (PKAcat) (Taylor et al., 2013). Activated PKAcat, glycogen synthase kinase 3β (GSK3β), and casein kinase (CK) phosphorylate full-length glioma-associated oncogene transcription factors Gli2 and Gli3 (GliFL). Phosphorylation and the following proteolytic cleavage convert Gli transcription factors into their repressed inactive form (GliR) (Wang and Li, 2006; Mukhopadhyay and Rohatgi, 2014; Cohen et al., 2015; Niewiadomski et al., 2019).

In the Shh on state, Hh ligand binds to Ptch1. Ptch1 then exits the ciliary membrane, and its inhibiting effect on Smo is lifted. Smo and Gli1 translocate to the cilium and are transported to the ciliary tip in an IFT-dependent manner. Accumulation of Smo at the tip results in a dissociation of the suppressor of fused (SuFu) from GliFL. This is followed by phosphorylation and the subsequent formation of Gli transcriptional activator (GliA) (Wang et al., 2010; Chen et al., 2011; Niewiadomski et al., 2014). In addition, GPR161 exits the cilium, the cAMP level decreases, and PKA activity is reduced, ultimately leading to GliR downregulation (Singh et al., 2015). Thus, the ratio of active GliA to inactive GLiR is shifted toward GliA and, hence, to induction of nuclear Hh target genes (Rohatgi et al., 2007; Humke et al., 2010; Chen et al., 2011; Robbins et al., 2012; Santos and Reiter, 2014), which are involved not only in ciliogenesis but also in embryonic development and tissue homeostasis (Lettice et al., 2003; Jacob et al., 2011; Robbins et al., 2012).

In this study, our aim was to understand disease-related mechanisms induced by the missense mutation (MM) A375V. In a first attempt, changes in protein–protein interaction were investigated by affinity purification to identify candidates that might be involved in A375V dysfunction. Differences in the abundance of protein interactors hinted at a disturbed interaction pattern. Second, the paralog-specific role of wildtype and mutant TTC30A and B was investigated. Therefore, single-knockout, double-knockout, and TTC30A/B wildtype rescue cells generated in a previous study (Hoffmann et al., 2022), as well as newly created TTC30A/B A375V mutant rescue cells, were analyzed. Loss or mutation of TTC30A led to a specific transport defect of Shh signaling components and cAMP-related PKA substrate localization, which was not seen upon TTC30B disturbance. The data presented here integrate the paralog TTC30A as an essential component of the Shh pathway, whereas TTC30B dysfunction might reflect a rather subtle Shh-dependent phenotype.

## 2 Methods

### 2.1 Generation of mutant and fluorescence cell lines

TTC30A and/or TTC30B knockout cell lines (KO, hTERT-RPE1 (CRL-4000, and ATCC)) generated before were used for stable rescue line generation. The detailed creation of p.G12VfsX50 (referred to as TTC30A KO), p.G6AfsX28 (referred to as TTC30B KO), and the combined p.G12VfsX50/p.G6AfsX28 (referred to as TTC30A/B double KO) is described elsewhere (Hoffmann et al., 2022). For rescue experiments, hTERT-RPE1 TTC30A/B double-KO cells were stably transfected with TTC30A or TTC30B wildtype constructs (TTC30A/B pDEST (Invitrogen, United States) modified with N-terminal Strep/FLAG-tag (by CJ Gloeckner) as well as constructs harboring a TTC30A/B A375V mutation (Gloeckner et al., 2009; Hoffmann et al., 2022). These missense constructs were generated via site-directed mutagenesis based on the Strep/FLAG-tagged TTC30A/B wildtype constructs. For investigation of Shh signaling, hTERT-RPE1 wildtype cells were stably transfected with Ptch1 fluorescence plasmids (pPtc1-YFP; Addgene, Watertown, MA, United States). Neomycin resistance (NeoR) encoded by the rescue/fluorescence construct was used for antibiotic selection of stably transfected cells. Cells were treated for 4 weeks with 0.4 mg/mL geneticin disulfate-supplemented (G418, Carl Roth, Germany) DMEM. All generated TTC30A/B wildtype, A375V mutant, KO, and Ptch1 fluorescent cell lines were treated with Dulbecco’s Modified Eagle’s Medium (DMEM, Sigma-Aldrich, St. Louis, MO, United States), supplemented with 10% fetal bovine serum (Sigma-Aldrich, United States) and 0.5% penicillin/streptomycin (Thermo Fisher Scientific, Waltham, MA, United States), and incubated at 37°C and 5% CO_2_.

### 2.2 Immunofluorescence staining

Control and KO hTERT-RPE1 cells were phenotypically analyzed as described by Hoffmann et al. (2022) and in this study together with TTC30A/B wt and A375V rescue cells used for further localization studies. To induce cilia formation, confluent cells were serum deprived for 3 days. To activate the Shh signaling pathway, 52.65 µL Smoothened agonist (SAG; 1:1000, AbMole Bioscience, Houston, TX, United States) was added to 1 mL DMEM (final concentration of 100 nM) 24 h prior to fixation (4% PFA, Morphisto, Offenbach, Germany). This was followed by permeabilization with 0.3% PBST and blocking with 10% normal goat serum in 0.1% PBST. The cells were incubated with a primary antibody solution and subsequently with a fluorescent secondary antibody solution (Alexa Fluor 488/568, 1:350; Invitrogen, Waltham, MA, United States). Finally, all samples were mounted with Fluoromount-G (Invitrogen, United States) and examined via fluorescence microscopy.

Images were captured using a Zeiss Axio Imager Z1 ApoTome microscope (Carl Zeiss Microscopy GmbH, Munich, Germany). The setup includes an AxioCam MRm camera as well as 40× (NA 1.3) and 63× (NA 1.4) oil immersion objective lenses. Images were acquired as Z-stacks and processed using Zeiss ZEN 3.0 Blue Edition (Carl Zeiss Microscopy GmbH, Germany).

### 2.3 SDS-PAGE and western blot

SDS-PAGE and western blot were performed in this study to investigate the total protein level of PKAcat (1:1000; Santa Cruz, Germany) and phospho-PKA substrate (RRXS*/T*, 1:500/1:1000; Cell Signaling Technology, Danvers, MA, United States). Therefore, to inhibit the Shh signaling pathway, 0.5–2 μL/mL forskolin (4 μg/μL; Sigma-Aldrich, United States) was added 1 h prior to cell lysis (final concentration of 5 µM). For SDS-PAGE, an 8% Tris-glycine-based separation gel and running buffer was used. Full wet tank blotting (Bio-Rad, Hercules, CA, United States) was followed by 5% BSA block as well as primary and secondary antibody (goat α rabbit/mouse antibodies, 1:10000; Jackson ImmunoResearch, Philadelphia, PA, United States) incubation. Membranes were treated with ECLplus (Thermo Fisher Scientific, United States), and images were taken using the Fusion FX7 imaging system (Vilber, Collégien, France).

### 2.4 Real-time quantitative PCR

RT-qPCR was performed in this study to investigate the mRNA expression level of Gli1. To activate the Shh signaling pathway, 52.65 µL Smoothened agonist (SAG; 1:1000, AbMole Bioscience, Houston, TX, United States) was added to 1 mL DMEM (final concentration of 100 nM) 24 h prior to cell lysis. RNA was isolated by using TriFast (VWR, Radnor, PA, United States) and chloroform and precipitated with isopropanol. The final concentration was measured with a NanoDrop^®^ ND-1000 spectrophotometer (Peqlab, Erlangen, Germany) and adjusted to 0.1μg/μL. Then, cDNA was synthesized with M-MLV reverse transcriptase (Bio-Rad, Hercules, CA, United States) and analyzed with SYBR Green Supermix (Bio-Rad, Hercules, CA, United States) in an RT-qPCR cycler (Bio-Rad, Hercules, CA, United States).

### 2.5 Affinity purification

Comparative interactome analysis by Strep-tag-based affinity purification was carried out as described in earlier studies (Boldt et al., 2009; Boldt et al., 2011). In brief, HEK293T cells were transfected with Strep/FLAG-tagged constructs containing either the TTC30A/B wildtype sequence or the mutated A375V variant. Strep/FLAG-tagged RAF1 was used as cilia-independent control. At full confluency, cells were harvested and lysed (lysis buffer includes 1x TBS, 0.5% Nonidet-P40, PI2/3, and PIC) in an end-over-end shaker at 4°C for 30 min. After lysis, the protein concentration was measured using the Bradford assay. Identical amounts of protein for each sample were incubated with Strep-Tactin Superflow (IBA, Göttingen, Germany) for 1.5 h, followed by three washing steps and elution of bound protein with Strep elution buffer (IBA, Germany). A methanol–chloroform-based protein precipitation was performed with subsequent trypsin digestion at 37°C overnight. The digested proteins were desalted via stop-and-go extraction tips (Thermo Fisher Scientific, United States) and prepared for mass spectrometry (MS) analysis.

### 2.6 Mass spectrometry

For LC–MS/MS analysis, an UltiMate 3000 nano-RSLC was coupled to a Fusion by a nanospray ion source. Prepared peptide mixtures were loaded onto a nanotrap column (µ-Precolumn 300 µm i.d. × 5 mm, packed with Acclaim PepMap100 C18, 5 μm, 100 Å; Dionex, Sunnyvale, CA, United States). Injection was conducted with a flow rate of 30 μL/min in 98% of buffer C (0.1% TFA in HPLC-grade water) and 2% of buffer B (80% ACN and 0.08% formic acid in HPLC-grade water). After 3 mins, peptides were eluted and separated on an analytical column (75 μm × 25 cm, packed with Acclaim PepMap RSLC, 2 μm, 100 Å; Dionex, United States) at a flow rate of 300 nL/min with a linear gradient from 2% up to 30% of buffer B in buffer A (2% ACN, 0.1% formic acid) for 82 min after an initial step of 3 min at 2% buffer B. The remaining peptides were eluted with a steep gradient (30%–95% in 5 min) followed by 5 min at constant 95% of buffer B before the gradient was decreased rapidly in 5 min to 2% of solvent B for the final 20 min. In the data-dependent analysis, full-scan MS spectra were measured on the Fusion in a mass-charge range from m/z 335–1500 with a resolution of 70,000. The ten most abundant precursor ions were selected with a quadrupole mass filter if they exceeded an intensity threshold of 5.0 e4 and were at least doubly charged for further fragmentation using higher-energy collisional dissociation (HCD) followed by mass analysis of the fragments in the iontrap. The selected ions were excluded for further fragmentation in the following 20 s. Max Quant software 1.6.1.0 was used (Cox and Mann, 2008) for label-free quantification (Supplementary Table S1) with the current SwissProt database and Perseus software 1.6.2.3/1.6.5.0 for data and statistical analysis (Student’s t-test; significance A) (Tyanova et al., 2016). Data are available from ProteomeXchange with identifier PXD044183.

## 3 Results

### 3.1 TTC30A mutation A375V leads to decreased IFT57 interaction

Missense mutations (MMs) might lead to a decrease in protein stability and prevent the formation of functional relevant protein complexes. Changes in the protein–protein interaction (PPI) pattern are used to determine candidates that are involved in wildtype and disease-relevant mechanisms (Boldt et al., 2016; Leonhard et al., 2023). To investigate the influence of TTC30 A375V mutation on PPI, a comparative mass spectrometry analysis was performed. Therefore, HEK293T control cells were transiently transfected with an overexpression vector containing a Strep/FLAG-tag and either TTC30A or B wildtype sequence as control or the mutated A375V variant (Gloeckner et al., 2009; Du et al., 2019).

**FIGURE 1:**
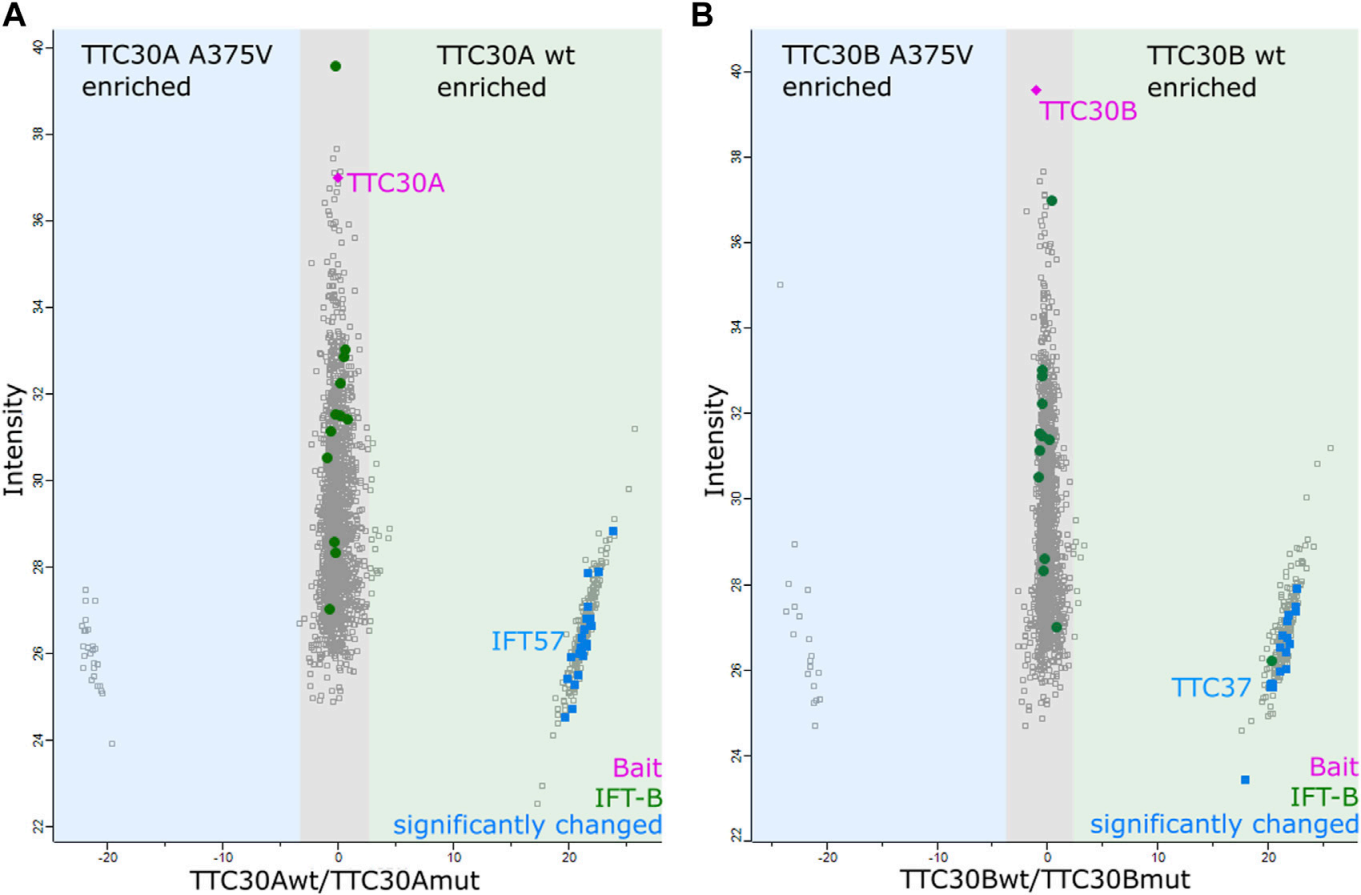
Detection of decreased protein interaction in mutated TTC30A/B by mass spectrometry analysis. The scatter plots show the distribution of all proteins identified for TTC30A **(A)** as well as TTC30B **(B)**. The *x*-axis depicts the log2 ratios, and the *y*-axis shows the log2 intensities. The bait proteins TTC30A and B are shown in magenta, and IFT complex B proteins are shown in green. Proteins showing an increased binding to TTC30A/B wt are on the right compared to TTC30A/B A375V on the left. Possible protein interactors that were significantly enriched for TTC30A/B wt (significance A < 0.05; permutation-based FDR <0.05) are shown in blue.

Cells were lysed, followed by a Strep-tag-based affinity purification, as carried out in earlier studies (Gloeckner et al., 2009; Hoffmann et al., 2022). After protein digestion and purification, quantitative mass spectrometry was performed. MaxQuant software and its implemented label-free quantification (LFQ) algorithm were used for the identification and quantification of proteins (Cox and Mann, 2008). This algorithm uses a pairwise matrix of peptides identified and quantified across all samples to generate a normalization factor based on a minimum of two peptides between two samples; then, the whole profile is rescaled to the cumulative intensity across samples to preserve the total summed intensity for a protein over all samples (Cox et al., 2014). These LFQ intensities were then statistically analyzed using Perseus (Tyanova et al., 2016). All identified proteins were filtered, removing proteins only identified by site, reversed peptide sequences, and potential contaminants. The groups of six biological replicates for each condition (control, TTC30A or B wt, and TTC30 A375V A or B) were filtered for a minimum of four valid values. Non-valid values, which were left, were replaced by 0. The median value of the LFQ intensities in one group was calculated, and the ratio was determined. First, TTC30A or B wt were compared to control transfected cells to detect the wt PPI in this experiment. Proteins were considered as specific interactors when they were Student’s t-test (permutation-based FDR <0.05) and significance A positive (Benjamini–Hochberg FDR <0.05). Second, TTC30A- or B-specific PPIs were investigated in the related A375V mutant (Figure 1). Proteins with significantly increased abundancy in cells transiently transfected with wildtype TTC30A/B and significantly decreased abundancy in cells transiently transfected with mutated TTC30A/B were considered proteins with decreased interaction.

The bait signal for either TTC30A or TTC30B and the related mutant protein were detected with comparable abundance, confirming reliable assay performance and bait protein stability. Compared to the control, 106 proteins were enriched with TTC30A, and 74 proteins were enriched when TTC30B was used as bait (Supplementary Table S2), with 10 IFT-B proteins being stably present in A and B wt samples (IFT88, −74, −172, −57, −46, −22, −56, −80, −52, and −81). Regarding wt and mutated TTC30A/B, almost every IFT-B complex protein was equally present in all groups, indicating that A375V has no influence on the overall complex composition. However, the signal of IFT57, part of the IFT-B2 subcomplex, was significantly decreased in TTC30A A375V cells, hinting toward impairment of this specific PPI. In total, 34 proteins showed reduced interaction due to A375V missense mutation when comparing wt and mutant TTC30A (Supplementary Table S2), and 21 proteins in cells transfected with mutant TTC30B (Supplementary Table S2). These were further confined by matching them with the SYSCILIA Gold Standard highlighting ciliary relevant proteins (Boldt et al., 2016). The 21 and 15 SYSCILIA Gold Standard proteins showed significantly reduced protein abundance in mutant TTC30A and TTC30B, respectively (Table 1). Interestingly, only two of the decreased PPIs were found in both datasets for TTC30A and TTC30B, RNPS1 and SMAP.

**TABLE 1:** Decreased TTC30A protein interactions due to A375V mutation. An affinity purification was performed with HEK293T cells transiently transfected with either Strep/FLAG-tagged TTC30A wt or Strep/FLAG-tagged TTC30A A375V. The protein names of identified interactors with ciliary relevance (Syscilia) and their ratio (TTC30Awt/TTC30A A375V) after comparative analysis are listed according to their -log2 median *p*-values of six biological replicates. All proteins shown were defined as interactors when compared to control and significantly decreased in TTC30A A375V when compared to TTC30A wt (significance A < 0.05, permutation-based FDR <0.05).

A	Proteins	Ratio TTC30A wt/A375V	*p*-value	B	Proteins	Ratio TTC30B wt/A375V	*p*-value
	RAP1GDS1	21.268	15.9578		SMU1	21.1202	16.7305
	PAWR	21.1571	16.3921		AKR1A1	21.9423	3.25847
	SLC35F6	19.6741	3.26108		SMAP	21.3886	3.26505
	ABCD1	20.4975	3.26545		FAM162A	21.924	3.16695
	RAB21	21.5211	3.26127		NT5DC1	21.6923	3.12479
	ATXN2	20.9478	3.24142		INTS3	17.9108	1.98927
	SPNS1	21.8258	3.26729		SLC9A3R1	20.4019	1.99255
	SMAP	21.8596	3.26887		NFS1	21.1456	1.96761
	RNPS1	22.577	3.26902		TTC37	20.4085	1.99333
	NDUFB11	21.915	2.15614		SNRNP40	21.7892	1.99359
	CCDC22	19.8702	1.99343		OSBP	22.516	1.92822
	USMG5	23.8228	1.99371		RNPS1	22.6874	1.92897
	MRPS14	21.6745	1.99456		EIF2AK2	21.6629	1.75515
	HNRNPLL	20.2489	1.99458		GEMIN4	22.5613	1.73741
	PPP1R8	20.1523	1.99461		GFM1	21.7937	1.72515
	VPS11	20.8415	1.99473				
	PDXDC1	21.1021	1.87951				
	GRWD1	21.6523	1.86699				
	CDC27	21.3169	1.70501				
	HEATR3	20.9969	1.68596				
	IFT57	21.4061	1.61882				

### 3.2 Localization of IFT57 is unaffected by TTC30 mutation A375V

**FIGURE 2:**
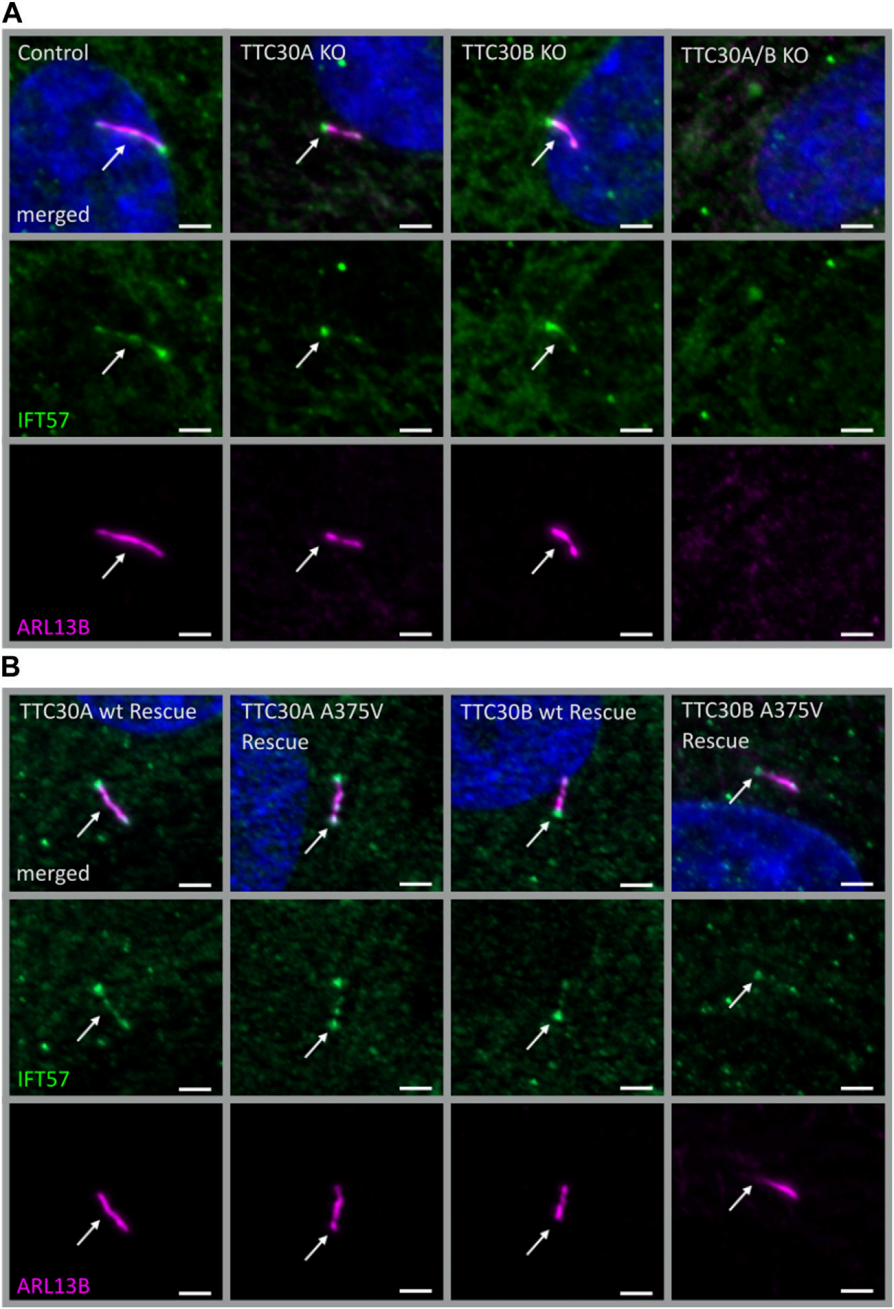
Similar localization of IFT57 in TTC30A/B wt, KO, and rescue cells. Fluorescent light microscopy images of hTERT-RPE1 control, TTC30A, TTC30B, and TTC30A/B double-KO cells (from left to right) **(A)**. Additionally, hTERT-RPE1 TTC30A wt, TTC30A A375V, TTC30B wt, and TTC30B A375V rescue cells are depicted **(B)**. Cells were stained for ARL13B (magenta) and IFT57 (green), and co-localization is shown in white. The scale bar measures 2 µm.

The next step was to investigate whether the reduced TTC30A–IFT57 interaction shown by PPI analysis affects the ciliary localization of IFT57. In addition to the mass spectrometry data shown here, the overlapping phenotype of shortened cilia in TTC30A or B knockout (KO) and upon knockdown of IFT57 hints toward a common function of these two IFT-B proteins (Kramer-Zucker et al., 2005; Hoffmann et al., 2022). Here, the effect of depletion of either TTC30A or B on IFT57 was investigated. In addition, the rescue potential of TTC30A or B wt and the respective A375 variant was analyzed in the TTC30A/B double-KO background. Control, TTC30A/B single-KO, and double-KO hTERT-RPE1 cells, which were already generated and described in a previous study, were used (Hoffmann et al., 2022) (Figure 2A). For mutant analysis, TTC30A/B double-KO cells were stably transfected with either TTC30A or B wt or the respective A375V variant (Figure 2B), and all cells were stained for ARL13B and IFT57. ARL13B localizes to the ciliary membrane and is, therefore, frequently used for ciliary length measurements (Dummer et al., 2016).

**FIGURE 3:**
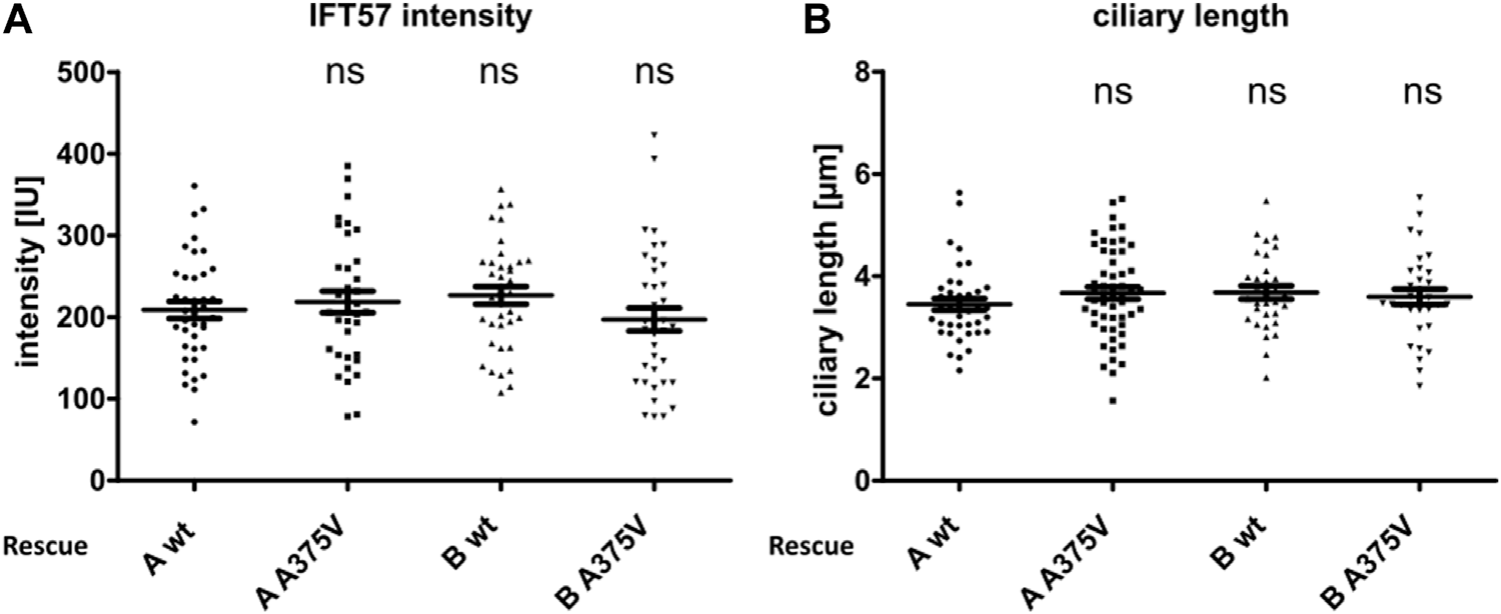
Ciliary intensity of IFT57 and ciliary length were comparable in TTC30A/B wt and A375V rescue cells. The IFT57 fluorescence intensity **(A)** and the ciliary length **(B)** were measured using ARL13B as the cilia marker. In the scatter dot plots, results gained in two independent experiments are shown, with each dot representing one cilium. For statistical analysis, the corrected total cell fluorescence **(A)** and the mean **(B)** were calculated. *p*-values above 0.05 were determined not significant (ns). Error bars represent the s.e.m.

In all cell lines analyzed except the double-KO, which lack cilia, IFT57 was detected in the cilium (Figure 2). To determine if IFT57 localization to the cilium differed between wt and A375V rescue cells, intensity was measured in the generated stable rescue cell lines (n_TTC30AwtRescue_ = 39; n_TTC30BwtRescue_ = 37; n_TTC30A-A375Rescue_ = 36; n_TTC30B-A375Rescue_ = 37). The average intensity was 208.8 IU ± 10.40 IU (in TTC30A wt rescue cells) and 226.5 IU ± 10.86 IU (in TTC30B wt rescue cells). In TTC30A/B double-KO cells rescued with mutant TTC30A or TTC30B, average intensity was 218.5 IU ± 13.05 IU (in TTC30A A375V rescue cells) and 197.1 IU ± 14.04 IU (in TTC30B A375V rescue cells). The IFT57 signal intensity in A375V rescue cells was not significantly decreased compared to wt rescue cells (Figure 3A). Regarding ciliary length, the average in TTC30 wt rescue cells was 3.449 µm ± 0.1132 µm, in TTC30A A375V rescue cells 3.671 µm ± 0.1212 µm, in TTC30B wt rescue cells 3.680 µm ± 0.1309 µm, and in TTC30B A375V rescue cells 3.595 µm ± 0,1512 µm (n_TTC30AwtRescue_ = 41; n_TTC30AmutRescue_ = 54; n_TTC30BwtRescue_ = 32; n n_TTC30BmutRescue_ = 32). Hence, the average ciliary length measured in TTC30A/B wt was comparable to that in TTC30A/B A375V rescue cells (Figure 3B). These results suggest that even though there were significant changes in binding of IFT57, TTC30A/B KO or A375V mutation did not influence the localization of IFT57 and, subsequently, ciliary length.

### 3.3 PKA-mediated downregulation of the Shh signaling pathway is dependent on TTC30A

Based on the data presented before, reduced PPI was described, which support a better understanding of the molecular function of TTC30 paralogs and related mutations. However, no direct link to Shh signaling could be found, which would help understand defects leading to synpolydactyly in individuals carrying A375V MM. (Du et al., 2019). As overexpression might mask specific PPIs of interest, data gathered in a previously published study providing a paralog-specific interactome on the endogenous level were re-assessed regarding Shh signaling relevant proteins (Hoffmann et al., 2022).

In addition to overlapping proteins belonging to the IFT-B1 or IFT-B2 subcomplex, a total of 44 proteins were found to be significantly enriched with TTC30A, and 47 proteins were identified with TTC30B (Hoffmann et al., 2022). Intriguingly, PRKACA was one of the significant interactors of TTC30A. This protein is the catalytic subunit α of the protein kinase A, which is a known key regulator of Shh and described as a part of the ciliary proteome (Mick et al., 2015).

These interactome data suggested a paralog-specific role in Shh signaling for TTC30A and further hint toward a TTC30A-dependent regulation of PKA activity. PKA is a cAMP-dependent protein kinase A and has several functions in glycogen, sugar, and lipid metabolism (London and Stratakis, 2022). Moreover, this kinase is a negative regulator of the Shh pathway and essential for the phosphorylation of the Gli transcription factors (Wang and Li, 2006; Mukhopadhyay et al., 2013; Niewiadomski et al., 2019). Forskolin inhibits the Shh pathway by increasing ciliary cAMP level and, subsequently, the level of phosphorylated PKA (pPKA), resulting in the increased phosphorylation of PKA substrates (Ding and Staudinger, 2005). To investigate the TTC30-related regulation of PKA activity, hTERT-RPE1 control, TTC30A, and TTC30B KO cells were serum-starved, followed by treatment with DMSO or forskolin (5 µM) for 1 h.

**FIGURE 4:**
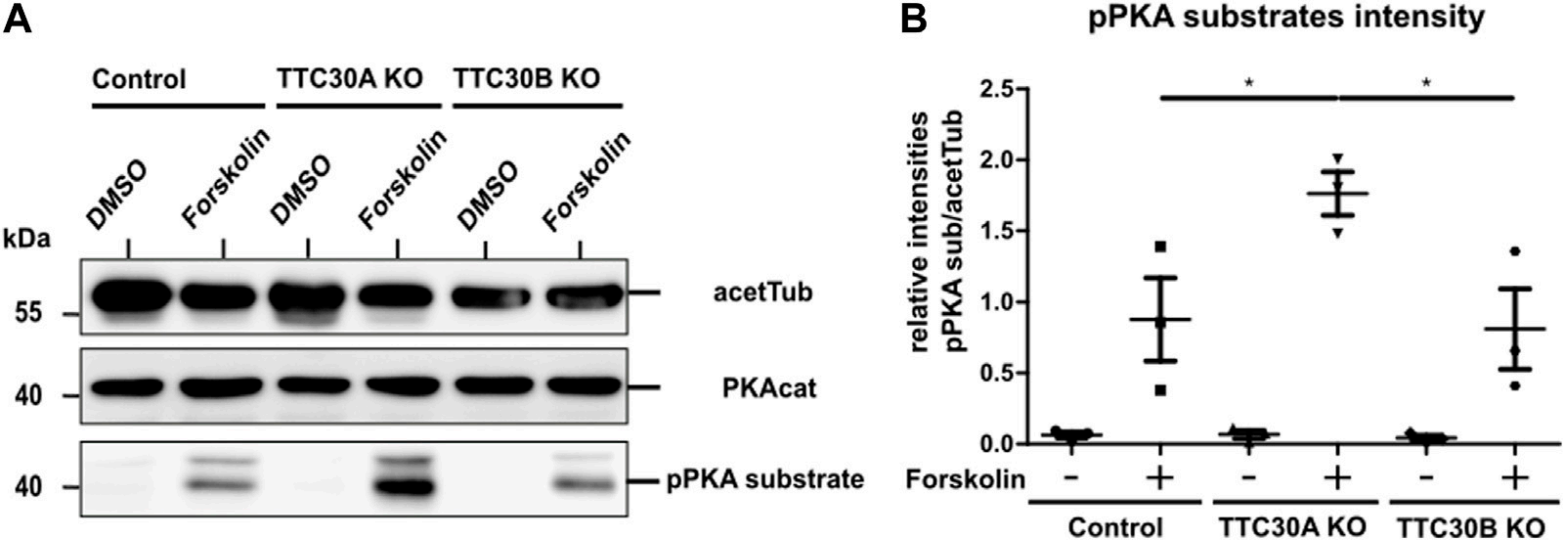
Protein level of pPKA substrates are elevated in forskolin-treated TTC30A KO cells. **(A)** Total protein levels of the PKA catalytic subunit, phosphorylated PKA substrates, and acetylated tubulin as loading control were detected by western blot after treatment with DMSO or forskolin (5 µM) 1 h prior to cell lysis **(B)**. Protein levels were quantified by measuring their intensity. These values were normalized to the loading control (acetylated tubulin). Resulting ratios of pPKA substrate intensity were statistically analyzed for all six conditions (n = 3) using a one-way ANOVA followed by Tukey’s test. *p*-values below 0.05 were deemed significant and are represented by *. Error bars represent the s.e.m.

After cell lysis, SDS-PAGE and western blot were performed to visualize protein level differences of PKA catalytic subunits and the phosphorylated PKA substrate. The antibody applied against PKAcat targeted all three different subunits α, β, and γ. The phospho-site-specific antibody targeted PKA substrates containing a phospho-Ser/Thr residue with arginine at the −3 and −2 positions (Montminy, 1997). Acetylated tubulin was used as the loading control. Protein levels of PKAcat were comparable in all samples. Treatment with forskolin resulted in increased pPKA substrate levels in all three cell lines, proving the activating effect on protein kinase A in the hTERT-RPE1 cell model used in this study (Figure 4A). For further analysis, we performed a one-way ANOVA comparing relative intensities gathered in three independent experiments (*n* = 3) (Supplementary Figure S1). Therefore, in all three different cell lines that were either treated with forskolin or DMSO, pPKA substrate intensities were normalized to acetylated tubulin. A statistically significant difference in mean relative intensity was found (F (5, 12) = [14.84], *p* = < 0.0001) in five groups. Tukey’s test for multiple comparisons revealed that the mean relative intensity was significantly different between the control (−) vs. TTC30A KO (+) (*p* < 0.001, 95% C.I. = [−2.545, −0.8531], control (+) vs. TTC30A KO (+) (*p* < 0.05, 95% C.I. = [−1.733, −0.04076], TTC30A KO (−) vs. TTC30A KO (+) (*p* < 0.001, 95% C.I. = [−2.542, −0.8494], TTC30A KO (+) vs. TTC30B KO (−) (*p* < 0.001, 95% C.I. = [0.8738, 2.566], and TTC30A KO (+) vs. TTC30B KO (+) (*p* < 0.05, 95% C.I. = [0.1079, 1.800] (Figure 4B). Significantly, in forskolin-treated TTC30A KO cells, the signal intensity of phosphorylated PKA substrates was severely increased compared to that in control and TTC30B KO cells. The signals of the control and TTC30B KO cells were comparable.

This result hints toward a paralog-specific role of TTC30A for physiological PKAcat activation, which might depend on TTC30A–PRKACA interaction. To further understand the role of TTC30A, Shh signaling was investigated in more detail.

### 3.4 TTC30A KO presents distinct phosphorylated PKA substrate accumulation upon treatment with forskolin

Protein complex analysis proposed an interaction of TTC30A with PRKACA. An increased level of phosphorylated PKA substrates upon treatment with forskolin and subsequent inhibition of the Shh pathway in TTC30A KO cells suggested the functional relevance of this interaction. To investigate if TTC30A/B also influence the ciliary localization of phospho-PKA substrates, TTC30A or TTC30B single-KO and control hTERT-RPE1 cell lines were co-stained for ARL13B and phospho-PKA substrates. In addition, cells were treated with or without forskolin to influence the PKA activity.

**FIGURE 5:**
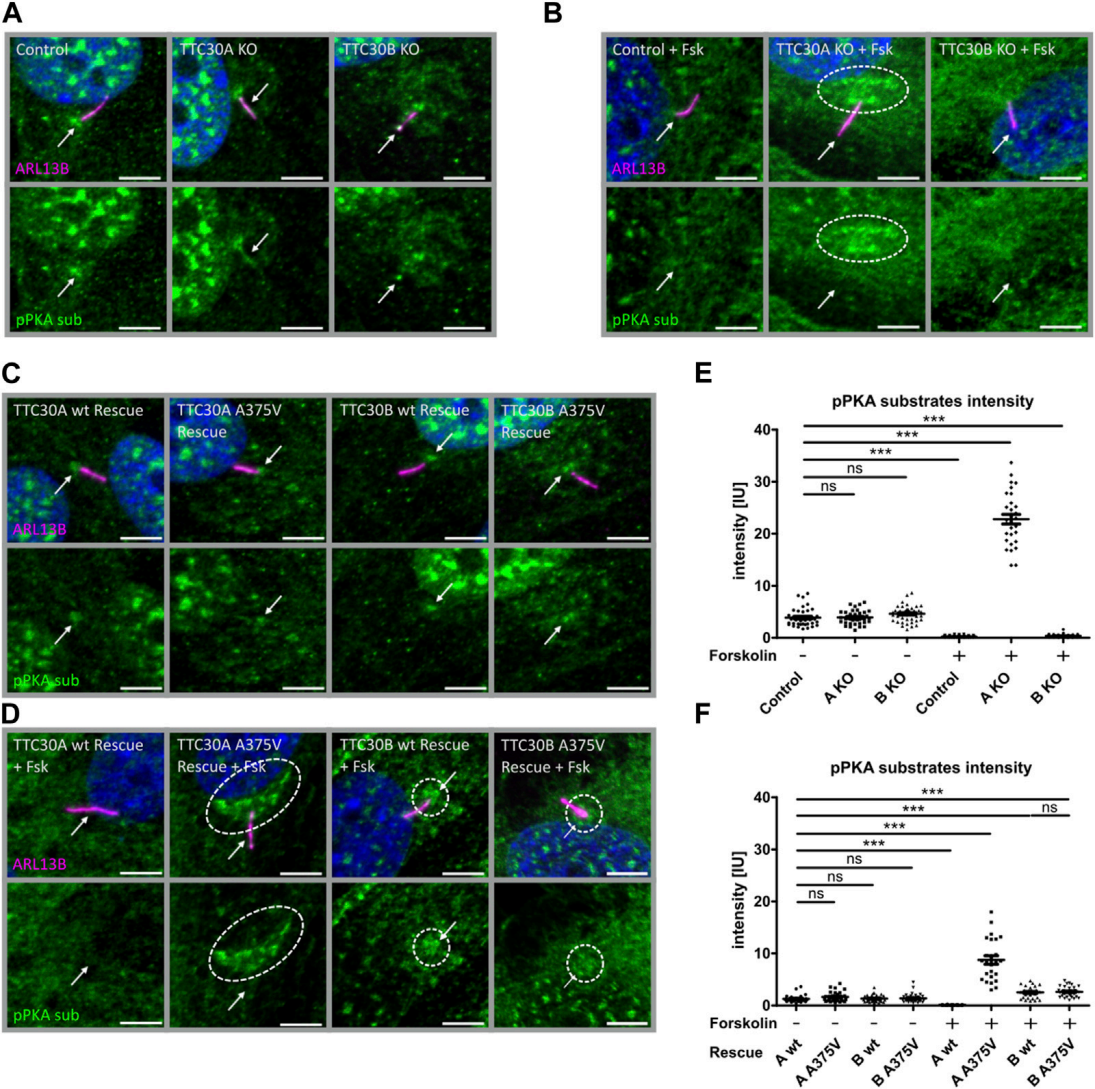
Phospho-PKA substrate accumulation detected in TTC30A KO and TTC30A A375V cells upon forskolin treatment. Fluorescent light microscopy images of hTERT-RPE1 control, TTC30A KO, and TTC30B KO (from left to right) are shown. Cells were either untreated **(A)** or treated with 5 µM forskolin for 1 hour **(B)**. Fluorescent light microscopy images of hTERT-RPE1 TTC30A wt, TTC30A A375V, TTC30B wt, and TTC30B A375V rescue cells (from left to right) are shown. Cells were either not treated **(C)** or treated with forskolin **(D)**. All cells were stained for ARL13B (magenta) and phospho-PKA substrates (green); DNA is marked in dark blue, and co-localization is shown in white. The scale bar measures 5 µm. Phospho-PKA intensity measurements were performed with TTC30A/B wildtype and knockout cells **(E)** as well as rescue cells **(F)**. Scatter dot plots are depicted, with each dot representing one cilium. For statistical analysis, relative intensity was calculated by measuring mean intensity followed by normalization to ARL13B. *p*-values below 0.001 are represented by ***, and those above 0.05 were determined not significant (ns). Error bars represent the s.e.m.

In untreated control, TTC30A KO and TTC30B KO cell phospho-PKA substrates were comparably localizing to the nucleus and basal body and with lower intensity to the cilium (Figure 5A). The measured mean intensities of the phospho-PKA signal localizing to the cilium and the basal body were normalized to their respective ciliary ARL13B mean intensities. Statistical analysis showed an average relative intensity of 3.878 IU ± 0.2734 IU in untreated control cells (n_control(−)_ = 39), 3.926 IU ± 0.2056 IU in untreated TTC30A KO cells (n_TTC30A KO(−)_ = 39), and 4.512 IU ± 0.2270 IU in untreated TTC30B KO cells (n_TTC30B KO(−)_ = 43; Figure 5E). Intriguingly, control and TTC30B KO cells treated with forskolin showed a decreased intensity of phospho-PKA substrates in the nucleus and at the basal body, whereas the localization to the cilium could not be distinguished from the background anymore (Figure 5B). Calculated relative intensities dropped to 0.2242 IU ± 0.02552 IU for forskolin-treated control cells (n_control(+)_ = 49) and to 0.3546 IU ± 0.04732 IU for treated TTC30B KO cells (n_TTC30B KO(+)_ = 39; Figure 5E). TTC30A KO cells treated with forskolin also showed lower to no localization of phospho-PKA substrates to the cilium and a reduced signal in the nucleus. However, in contrast to TTC30B KO, we could observe a distinct pattern of increased localization of phosphorylated PKA substrates at the basal body and the pericentriolar material (PCM) (Figure 5B) (Porpora et al., 2018). The average intensity of pPKA substrates strongly increased to 22.79 IU ± 0.9268 IU in forskolin-treated TTC30A KO cells (n_TTC30A KO(+)_ = 30; Figure 5E). This finding is most likely related to an elevated PKA activity and further confirms the paralog-specific function as well as influence of TTC30A on PRKACA.

To further validate these results, the ciliary localization of phospho-PKA substrates was also investigated in TTC30A/B wt and A375V rescue cells, again without or with forskolin treatment. We observed a localization of phosphorylated PKA substrates to the nucleus and basal body in all untreated cell lines, which confirms the previous findings in the control and TTC30A/B single-KO cells (Figure 5C). Their average relative intensities were 1.272 IU ± 0.1348 IU (n_TTC30AwtR(−)_ = 28), 1.630 IU ± 0.1689 IU (n_TTC30AmutR(−)_ = 31), 1.318 IU ± 0.1269 IU (n_TTC30BwtR(−)_ = 30), and 1.375 IU ± 0.1499 IU (n_TTC30BmutR(−)_ = 32). Nevertheless, overall average intensities in untreated stable rescue cell lines were reduced by the factor ∼2.5 compared to untreated control and TTC30A/B single-KO cell lines (Figure 5F). In wildtype TTC30A rescue cells, the previously described phenotype of forskolin-treated control and TTC30B KO cells showing reduced localization of phospho-PKA substrates to the nucleus, basal body, and cilium was also detected (Figure 5D). Similarly, the average relative intensity dropped to 0.1042 IU ± 0.0129 IU (n_TTC30AwtR(+)_ = 31) and was again reduced by the factor ∼2.5 compared to treated control and TTC30B KO cells (Figure 5F). Furthermore, the distinct pattern with an increased localization of phosphorylated PKA substrates at the PCM and basal body that could be observed in forskolin-treated TTC30A KO cells could also be described, but to a lesser extent, in TTC30A/B double-KO cells rescued with wildtype TTC30B as well as mutant TTC30A/B A375V (Figure 5D). Their average relative intensities were increased to 2.508 IU ± 0.2076 IU (n_TTC30BwtR(+)_ = 29) and 2.518 IU ± 0.1775 IU (n_TTC30BmutR(+)_ = 35; Figure 5F). Interestingly, the rare missense mutation A375V, introduced in TTC30A rescue cells, depicted a comparable phenotype as TTC30A KO cells upon forskolin treatment. The average relative intensity strongly increased to 8.755 IU ± 0.8210 IU (n_TTC30Amut(+)_ = 25) and was reduced by the factor ∼2.5 compared to treated TTC30A KO cells (Figure 5F). The observed systematic decrease of relative phospho-PKA intensity in the generated TTC30A/B wildtype and A375V mutant rescue cells most likely originates from stably transfecting TTC30A/B double-KO cells. These data support the finding of increased PKA activity, which was specifically related to TTC30A and, to a minor degree, TTC30B.

### 3.5 Loss of TTC30A inhibits the ciliary localization of Smoothened

**FIGURE 6:**
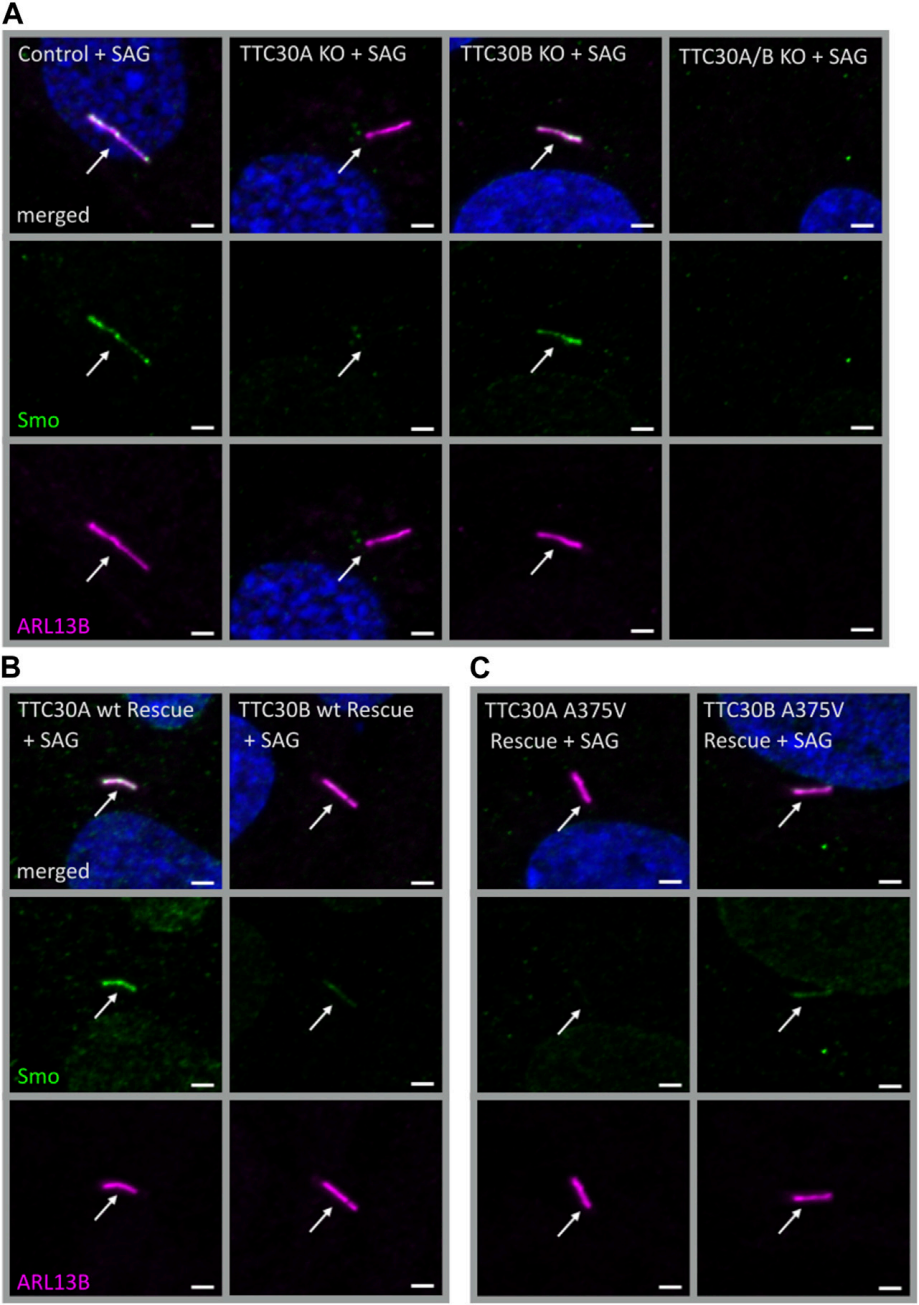
Smo localization was reduced in TTC30A KO cells and was rescued by TTC30A wildtype but not A375V mutant expression in TTC30A/B double-KO cells. Fluorescent light microscopy images of hTERT-RPE1 control, TTC30A, TTC30B, and TTC30A/B double-KO cells **(A)** (from left to right), TTC30A wt and TTC30B wt **(B)** (from left to right), and TTC30A A375V and TTC30B A375V rescue cells **(C)** (from left to right) are shown. All cells were treated with 100 nM SAG for 24 h. The cells were stained for ARL13B (magenta) and Smoothened (Smo; green), and co-localization is shown in white. The scale bar measures 2 µm.

In *D. melanogaster*, direct binding of PKA and Smo was shown (Jia et al., 2004; Li et al., 2014). Crosstalk between cAMP-dependent kinase signaling and Smo-related Shh signaling was described in other organisms as well (DeCamp et al., 2000; Chen and Jiang, 2013). The next step was to assess if solely PKA activity or if Smo-Ptch1 localization is affected in TTC30 KO cells as well. Initially, Smoothened (Smo), an early-stage key element of the Shh signaling pathway was investigated. Upon activation of the Shh pathway, Smo enters the cilium to trigger downstream effectors, which ultimately exit the cilium and activate target genes in the nucleus regulating cellular and cilia-related processes (Corbit et al., 2005; Niewiadomski et al., 2019). Hence, Smo localization was investigated in TTC30A or B KO, TTC30A/B double-KO, and control hTERT-RPE1 cell lines. Therefore, the Shh pathway was activated by treatment with the Smo agonist (SAG). The SAG initiates the activation of the Shh pathway and, subsequently, more Smo should accumulate at the cilium (Bragina et al., 2010). In control and TTC30B KO cells, Smo was normally localized, as seen by co-staining with ARL13B. However, this localization was not detected in TTC30A single-KO cells (Figure 6A). As TTC30A/B double-KO cells lack cilia, as shown before, no specific localization could be observed (Takei et al., 2018; Hoffmann et al., 2022). Ciliogenesis and Smo localization in TTC30A/B double-KO cells was restored by stably transfecting TTC30A wt DNA. Intriguingly, the stable transfection of TTC30B wt and A375V mutant DNA could only partially recover cilia assembly and Smo localization in TTC30A/B double-KO cells (Figure 6B). The ectopic expression of mutated TTC30A (A375V) was not sufficient to restore Smo, whereas TTC30A wt fully recovered Smo localization (Figure 6C).

**FIGURE 7:**
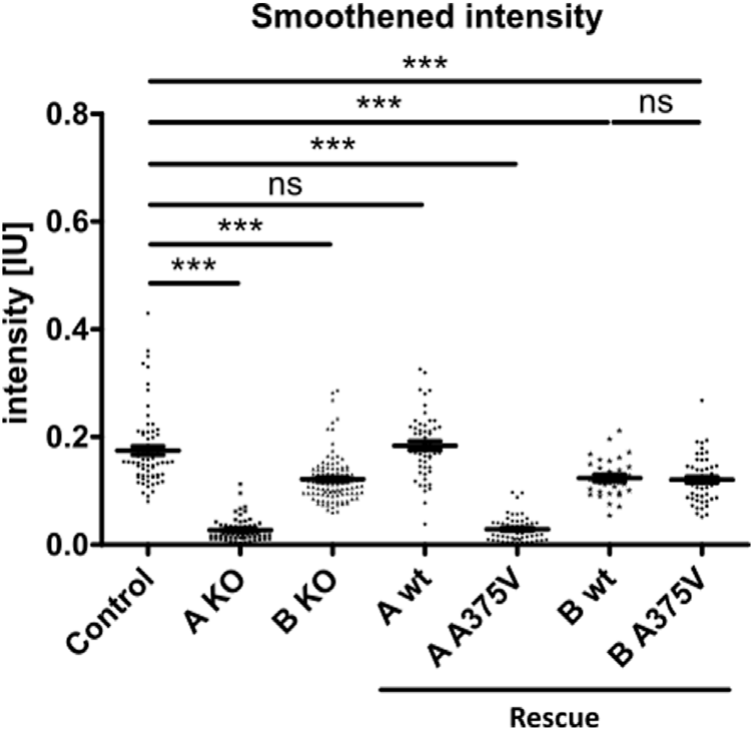
Smoothened intensity is reduced in TTC30A and B knockout as well as TTC30A A375V and TTC30B wildtype and A375V mutant rescue cells. In TTC30A/B control and single-KO as well as TTC30A/B wildtype and A375V mutant rescue cells, Smoothened intensity was measured and normalized to ARL13B intensity. In the scatter dot plots, results are shown with each dot representing one cilium. For statistical analysis, the relative intensity was calculated. *p*-values below 0.001 are represented by ***, and those above 0.05 were determined not significant (ns). Error bars represent the s.e.m.

This finding supports the hypothesis that TTC30A is involved in Shh pathway regulation. The loss of Smo localizing to the cilium could only be totally rescued by TTC30A wt. Transfection with TTC30A A375V did not recover this severe phenotype. Furthermore, the discovered mutation by Du *et al.* introduced in TTC30A could be directly linked to a loss of ciliary accumulation of Smo. However, the same A375V point mutation introduced in the TTC30B rescue construct did show this phenotype, but only to some degree. These initial results regarding Smo localization were further validated by measuring the mean intensity of Smo in TTC30A/B wildtype, single-KO, double-KO, and rescue cells (Figure 7). Again, mean intensity was normalized to ciliary ARL13B mean intensity. The average relative intensity was 0.1749 IU ± 0.008053 IU in control cells (n_control_ = 71). In TTC30A KO cells, the calculated relative intensity was clearly reduced to 0.02728 ± 0.002352 IU (n_TTC30A KO_ = 72). Interestingly, in TTC30B KO cells, a reduction of the average relative intensity to 0.1214 IU ± 0.004333 (n_TTC30B KO_ = 103) could be seen. Here, the effect was rather mild but also significant. Regarding rescue cells, the average relative intensity was 0.1839 IU ± 0.008152 IU (n_TTC30AwtR_ = 53) and, therefore, comparable to the control cells (Figure 7). Likewise, the average relative intensity of TTC30A A375V rescue cells was 0.02873 IU ± 0.003129 IU (n_TTC30AmutR_ = 54), which was comparable to Smo intensity in TTC30A KO cells. The average relative intensities in TTC30B rescue cells were 0.1235 IU ± 0.006528 IU (n_TTC30BwtR_ = 31) and 0.1204 IU ± 0.005563 IU (n_TTC30BmutR_ = 31), which were similar to each other and also to the average relative intensity in TTC30B KO cells (Figure 7). The overall quantification of Smo intensity resembled and verified the results of localization studies, but significantly, an additional mild reduction of Smo intensity in TTC30B KO cells was revealed.

The initial step of Shh signaling is Hh ligand binding to Ptch1, which then exits the cilium. Subsequently, Ptch1 relieves its inhibition of Smo, which in return enters the cilium and can affect downstream targets leading, among others, to the formation of Gli1 (Rohatgi et al., 2007). As Smo localization is dependent on ciliary exit of Ptch1, this re-localization of Ptch1 in TTC30A/B wildtype and knockout cells without or with Shh activation was investigated but appeared unaltered upon TTC30A loss (Supplementary Figure S3A). The SAG treatment itself does not affect the re-localization of Ptch1, but it activates the Shh pathway. This activated Shh pathway then leads to a degradation of Ptch1 mediated by ubiquitin (Yue et al., 2014; Desai et al., 2020; Yang et al., 2021).

The final outcome of Shh activation is the formation of Gli1. Hence, upon SAG-mediated Shh activation, the Gli1 level increases and should be affected due to the observed mis-localization of Smo in TTC30A/B single KO cells. Hence, mRNA expression levels determined by qPCR in SAG-treated and untreated TTC30A/B wildtype, KO, and rescue cells. Statistical analysis by one-way ANOVA [F(7,32) = (10.95), p = < 0.0001], followed by Tukey's test revealed that in TTC30A KO cells, upon SAG stimulation, Gli1 mRNA level was significantly reduced compared to that in control cells (p < 0.001, 95% C.I. = [0.0002802, 0.001176], which reflects the strong phenotype of Smo mis-localization. Furthermore, in TTC30B KO cells, a tendency to decreased Gli1 mRNA level is visible but not significantly different compared to the control, which resembles the mild Smo localization phenotype. The strong Gli1 mRNA expression observed in TTC30A/B wildtype and mutated A375V rescue cells that originate from the TTC30A/B double-KO cells is most likely driven by cilia-independent Shh signaling (Supplementary Figure S3B).

In conclusion, the data presented here suggest a paralog-specific function of TTC30A in Shh signaling. Smo localization and PKA activity were shown to be independent of Ptch1 function, but they do affect Gli1 formation and rely on TTC30A wildtype with mis-localization upon the loss or mutation of TTC30A. A possible involvement of impaired TTC30A–IFT57 interaction leading to synpolydactyly in A375V patients remains debatable.

## 4 Discussion

### 4.1 Patient A375V mutation leads to a decreased interaction of TTC30A with IFT57 and TTC30B with TTC37

Paralog proteins TTC30A and TTC30B share a remarkable resemblance regarding their sequence. Only a minor deviation (4.66% of the sequence) distinguishes TTC30A from TTC30B. In the double-KO background (Takei et al., 2018; Hoffmann et al., 2022), a paralog-specific rescue, which comprises either wildtype TTC30A or TTC30B, could equally recover TTC30-dependent cilia assembly and polyglutamylation to the level of single-KO cells (Hoffmann et al., 2022). Regarding these functions, TTC30A/B redundancy is extremely important for ciliogenesis and maintaining cilia function.

Despite these striking similarities in appearance and function, there was also evidence hinting at non-overlapping functions of TTC30A and TTC30B. In a Chinese pedigree, a rare TTC30B missense variant (A375V) was discovered and linked to Sonic hedgehog signaling (Shh) (Du et al., 2019). In *Xenopus tropicalis,* TTC30A was connected to ciliary chondrodysplasia with polycystic kidney disease. Intriguingly, they also assumed an involvement of Shh (Getwan et al., 2021). Using paralog-specific PPI investigation, we were able to show new and distinct interactions (Hoffmann et al., 2022).

In the study presented here, the influence of the A375V variant on wildtype TTC30A/B interactome was investigated (Du et al., 2019). TTC30B analysis revealed a reduced abundancy of TTC37 in the mutant (Table 1). TTC37 is a component of the SKI complex. It acts as a co-factor and regulator of the exosome-mediated decay of RNA. The malfunction of SKI is linked to the trichohepatoenteric syndrome (Kogel et al., 2022).

Interestingly, the IFT-B complex protein IFT57 binding to TTC30A was diminished for A375V in relation to wildtype TTC30A (Table 1). However, IFT57 localization and cilia length were not influenced in TTC30 A375V rescue cells, hinting toward normal IFT-B function in ciliogenesis in mutant cells.

Nevertheless, a disturbed TTC30A–IFT57 secondary interaction caused by A375V mutation would explain how this mutation is connected to Shh. IFT57 knockdown was shown to lead to disturbed Shh and polydactyly in mice (Houde et al., 2006). In addition, in humans, Shh dysregulation linked to reduced Gli1 induction and polydactyly was observed. However, a total loss of IFT57 ultimately leads to disrupted ciliary assembly and was shown to be lethal (Thevenon et al., 2016). However, no other direct link of TTC30A/B mutation and Shh signaling could be found in the study presented here using overexpression, indicating limitations of the assay based on transient overexpression (Gibson et al., 2013).

The IFT-B structural model recently described by Petriman et al. (2022) shows that TTC30 and IFT57 do not directly interact with each other. In the IFT-B1 complex, TTC30 is connected to IFT88 and IFT52. IFT88/52 then directly interact with IFT57 and IFT38, which belong to the IFT-B2 subcomplex (Katoh et al., 2016; Taschner and Lorentzen, 2016). Whether a minor conformational change in TTC30 due to A375V mutation (Du et al., 2019) would influence the binding pattern of IFT88/52 to IFT57 is up to speculation.

### 4.2 Loss of TTC30A function leads to PRKACA-dependent inhibition of the Sonic hedgehog pathway

Taken together, there are several indicators proposing an involvement of TTC30A/B in Shh. The discovered A375V mutation found in a Chinese pedigree was localized in the *TTC30B* gene. Shh signaling was disturbed due to the reduced Smo and Gli mRNA expression level, ultimately leading to synpolydactyly (Du et al., 2019).

Interactome data proposed an interaction of TTC30A with PRKACA Protein kinase A catalytic subunit α (PRKACA). PRKACA is a key regulator for Shh inhibition, which might help understand signaling misregulation observed by Du et al. (Taylor et al., 2013; Du et al., 2019). In the Shh off state, PKA phosphorylates full-length Gli2 and Gli3 (GliFL) transcription factors, which are then processed into their repressed forms (GliR). Upon Shh activation, GliFL is not processed to GliR but to its active form GliA. The ratio of GliA/GliR regulates the expression of target genes (e.g., Ptch1 and Gli1) (Cohen et al., 2015).

To investigate a possible impact of TTC30A KO on Shh, other pathway components were examined in the context of TTC30A/B single-KO cells and double-KO cells rescued with either wt or mutant TTC30A/B. In the Hh off state, PKA is active and phosphorylates its target substrates, which ultimately leads to an inhibition of Shh (Mukhopadhyay and Rohatgi, 2014).

The data presented here show that Smo does not enter the ciliary membrane in TTC30A KO or TTC30A A375V mutant cells, while simultaneously, the PKA activity increases. Similarly, but to a lesser extent, ciliary localization of Smo in TTC30B KO or TTC30B A375V mutant cells is reduced while PKA activity is only affected in TTC30B rescue cells. In 2021, Arveseth et al. (2021) showed that Smo is capable of recruiting and directly inhibiting PKA-dependent phosphorylation. Happ et al. were able to define a decoy substrate sequence of Smo, which blocks PKA-C activity (Happ et al., 2022). Our data indicate that TTC30A is involved in PKA inhibition in the Shh on state.

**FIGURE 8:**
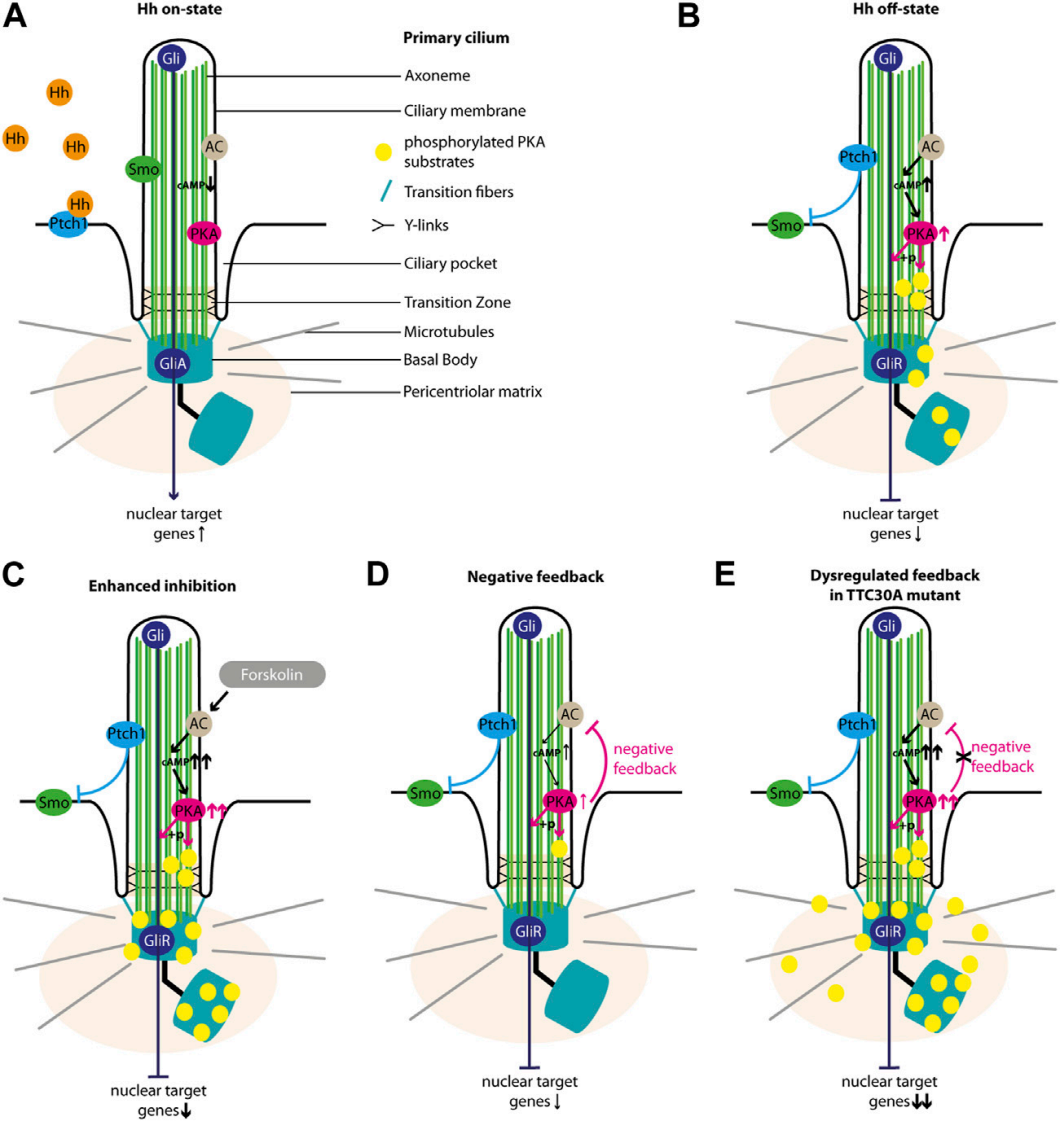
Overview of Sonic hedgehog signaling. The schematic view depicts the main regulators of Shh in the cilium. In the Hh on state, **(A)** Hh ligand binds to Ptch1, which exits the ciliary membrane and stops inhibiting Smo. Smo then enters the cilium, inhibits PKA activity, and is involved in the formation of GliA. In the absence of Hh ligand (Hh off state), **(B)** Ptch1 inhibits Smo. The AC is active and increases intraciliary cAMP level. CAMP increases the activity of PKA, which phosphorylates its substrates and is involved in the formation of GliR. Forskolin treatment **(C)** further increases the activity of AC and, hence, the cAMP level and PKA activity. PKA phosphorylates more substrates, and more GliR is formed. This strong Hh repression is counter-regulated by a PKA-mediated feedback loop **(D)** decreasing AC activity, cAMP level, PKA activity, the phosphorylation of PKA substrates, and GliR formation. In TTC30A KO and rescue TTC30A A375V cells, this PKA-mediated cAMP downregulation is disturbed **(E)**, leading to an overly active AC and PKA as well as an accumulation cAMP and phosphorylated PKA substrates.

We want to implement the data gathered here into the so far known regulation of the Shh pathway. In the Hh on state, Hh ligand binds to Ptch1, which leaves the ciliary membrane and its inhibition on Smo is lifted. Upon entering the ciliary membrane, Smo is involved in the dissociation of SUFU from GliFL transcription factors (Wang et al., 2010; Chen et al., 2011) and the inhibition of PKA activity (Arveseth et al., 2021; Happ et al., 2022). GliFL proteins can then be processed into their activated form, GliA, and the ratio of GliA/GliR is shifted toward GliA, which increases the activation of target genes in the nucleus (Figure 8A) (Niewiadomski et al., 2019). Localization studies of untreated control, TTC30A/B KO, and TTC30A/B double-KO cells rescued with either TTC30A/B wt or TTC30A/B A375V revealed the accumulation of phosphorylated PKA substrates at the BB and, more faintly, to the cilium (Figures 5, 8B). This resembles a basal level of PKA activity in the Hh off state (Moore et al., 2016). Smo cannot enter the cilium. The adenylyl cyclase (AC) is active and increases intraciliary cAMP level. Cyclic AMP phosphorylates the regulatory subunits of PKA, which then dissociate from the PKA complex and relieve the suppression of PKA catalytic subunits (Taylor et al., 2013). Subsequently, these can now phosphorylate GliFL and other PKA-specific substrates. GliFL is then cleaved into GliR, and the ratio of GliA/GliR now shifts toward GliR, which increases the repression of the target genes in the nucleus (Figure 8B) (Niewiadomski et al., 2019). Upon treatment with forskolin, we expected an increase in adenylyl cyclases 5 and 6 (AC5/6) activity and, therefore, elevated levels of cAMP, PKA activity, and phosphorylated PKA substrates, which was already shown based on the total protein level. This effect extends basal level Hh off state and leads to a shift of GliA/GliR ratio even more toward the repression of target genes (Figure 8C) (Ding and Staudinger, 2005; Vuolo et al., 2015; Moore et al., 2016). Interestingly, forskolin treatment revealed rather decreased ciliary phosphorylated PKA substrates in control, TTC30B KO, and wt TTC30A rescue cells (Figures 5, 8D). Here, a PKA-mediated feedback loop inhibits AC5/6 (Iwami et al., 1995; Murthy et al., 2002; Wangorsch et al., 2011). This negative regulation reduces intraciliary cAMP level and, thus, PKA activity (Murthy et al., 2002; Wangorsch et al., 2011; Cantero Mdel et al., 2015). This effect is further enhanced by the PKA-mediated activation of phosphodiesterase 3 (PDE3) (Murthy et al., 2002). Additionally, it was shown that PKA, as well as GSK3 and CK2, phosphorylates polycystin 2 (PKD2) (Cai et al., 2004; Streets et al., 2006; Cantero Mdel et al., 2015). Phosphorylated PKD2 increases the intraciliary Ca^2+^ level, which in turn also leads to a reduction of cAMP, probably due to the inhibition of Ca^2+^-sensitive AC5/6 (Nauli et al., 2003; Choi et al., 2011; Cantero Mdel et al., 2015). We hypothesize that this effect leads to a decline of pPKA substrates below the basal level of Hh off state (Figure 5). The consequence of this cAMP downregulation is a shift of GliA/GliR ratio by reducing GliR, followed by a lower repression of nuclear target genes (Figure 8D). This negative feedback loop and the subsequent PKA-mediated downregulation of AC, cAMP, and, finally, PKA itself is disturbed in TTC30A KO cells and TTC30A/B double-KO cells rescued with TTC30A A375V (Figure 8E). Hence, in these cell lines, PKA activity remains constantly increased, and its substrates are continuously phosphorylated and accumulate at the ciliary base and the PCM (Figure 5). Additionally, GliFL phosphorylation and cleavage to GliR are upregulated, altering GliA/GliR ratio strongly toward repression of the nuclear target genes (Figure 8E). Interestingly, it was shown that the deletion of *Pkd2* in mice led to an increased expression of phosphorylated PKA substrates (Choi et al., 2011), which strongly resembles the phenotype we describe in TTC30A KO and A375V TTC30A rescue cells.

In the study presented here, a functional relevance of TTC30A–PRKACA interaction was shown, confirming the involvement of TTC30A in Shh signaling. We demonstrated that the loss or A375V mutant TTC30A has an impact on Smo localization and the phosphorylation of PKA substrates. Such a strong Shh phenotype would lead to embryonic lethality, indicating that synpolydactyly seen in patients carrying the A375V mutation reflects a subtle symptom based on TTC30B mutation.

## Data Availability

The datasets presented in this study can be found in online repositories. The names of the repository/repositories and accession number(s) can be found in the article/Supplementary Material.

## Supplementary material

Supplementary Table S1 MaxQuant Settings for proteomic analysis—An overview of the MaxQuant settings used for quantitative analysis of acquired mass spectrometry data sets.
