# Supplementary figures and images for: Paralog-specific TTC30 regulation of Sonic hedgehog signaling
Source: Front Mol Biosci. 2023 Nov 23;10:1268722. doi: 10.3389/fmolb.2023.1268722 (PMC10701685; doi:10.3389/fmolb.2023.1268722)

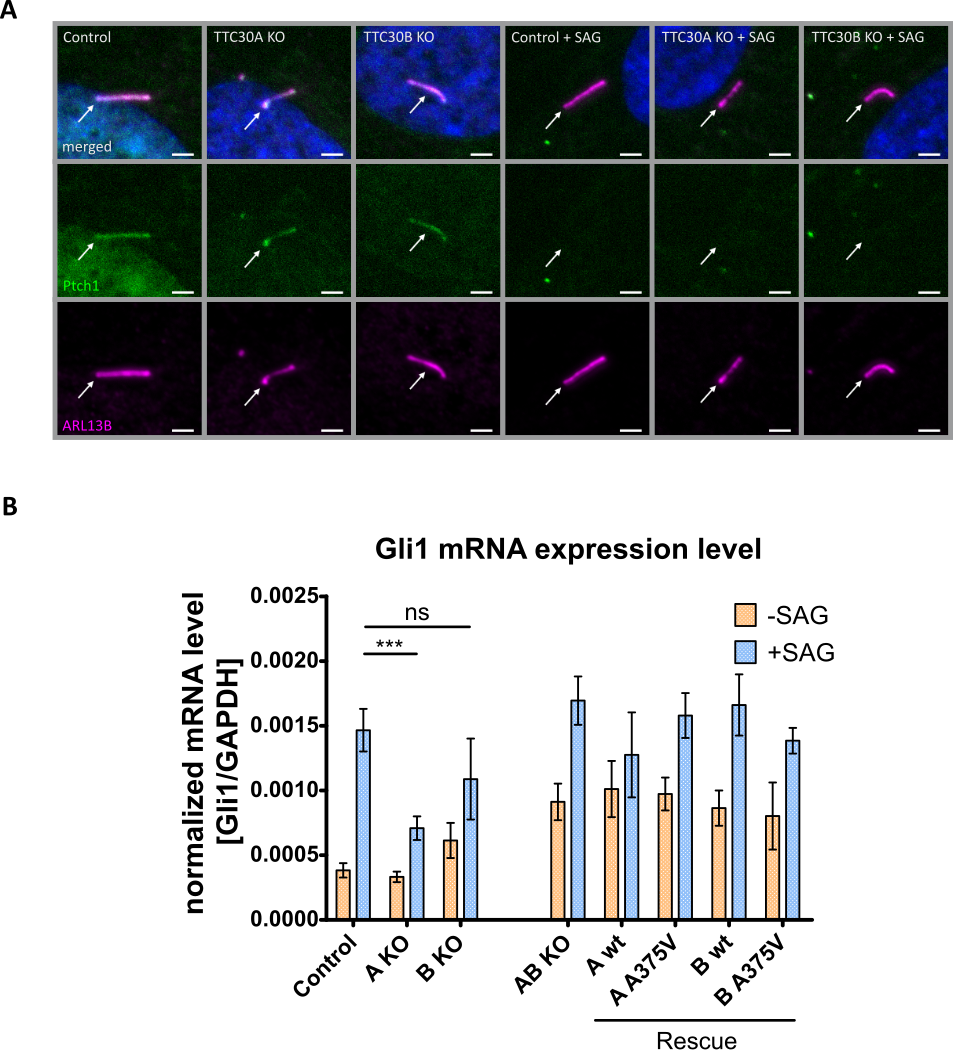

Supplement: Supplementary file 1 [file Image3.TIFF]

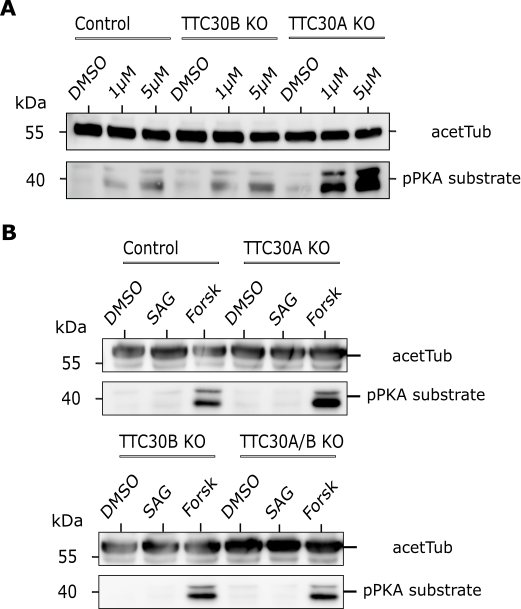

Supplement: Supplementary file 3 [file Image1.TIFF]

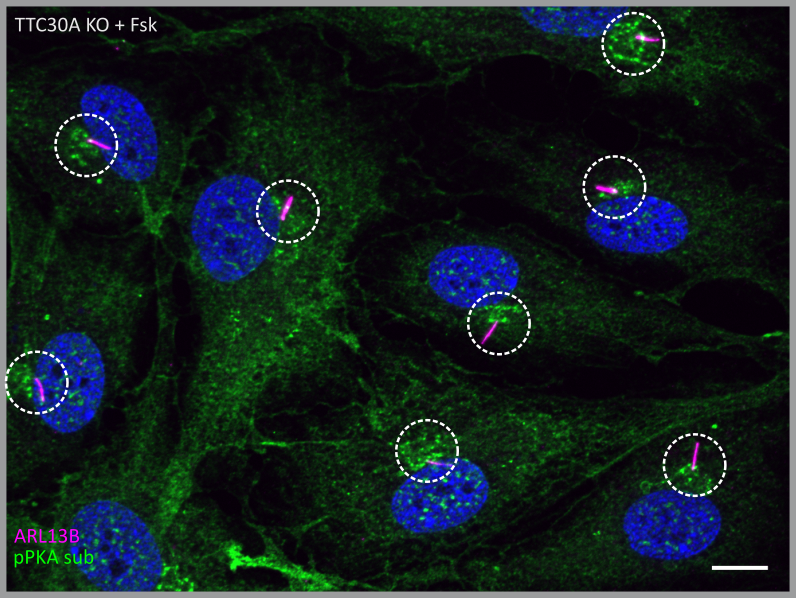

Supplement: Supplementary file 5 [file Image2.TIFF]
