# Supplementary material for: Paralog-specific TTC30 regulation of Sonic hedgehog signaling
Source: Front Mol Biosci. 2023 Nov 23;10:1268722. doi: 10.3389/fmolb.2023.1268722 (PMC10701685; doi:10.3389/fmolb.2023.1268722)
Supplement: Supplementary file 4 [file Table1.DOCX]

|  |  |
| --- | --- |
| Parameter Value  Version 1.6.1.0  User name mpcadmin  Machine name ADMIN-PC  Date of writing 05/22/2021 10:07:11  Fixed modifications Carbamidomethyl (C)  Include contaminants True  PSM FDR 0.01  Protein FDR 0.01  Site FDR 0.01  Use Normalized Ratios For Occupancy True  Min. peptide Length 7  Min. score for unmodified peptides 0  Min. score for modified peptides 40  Min. delta score for unmodified peptides 0  Min. delta score for modified peptides 6  Min. unique peptides 0  Min. razor peptides 1  Min. peptides 1  Use only unmodified peptides and False  Peptides used for protein quantification Razor  Discard unmodified counterpart peptides True  Label min. ratio count 2  Use delta score False  iBAQ False  iBAQ log fit False  Match between runs True  Matching time window [min] 0.7  Alignment time window [min] 20  Find dependent peptides False  Fasta file C:\Databases\Swissprot human 201911.fasta  Decoy mode revert  Include contaminants True  Advanced ratios True  Fixed andromeda index folder  Temporary folder  Combined folder location  Second peptides True  Stabilize large LFQ ratios True  Separate LFQ in parameter groups False  Require MS/MS for LFQ comparisons False  Calculate peak properties False  Main search max. combinations 200  Advanced site intensities True  LFQ norm for sites and peptides False  Write msScans table True  Write msmsScans table True  Write ms3Scans table True  Write allPeptides table True  Write mzRange table True  Write pasefMsmsScans table True  Write accumulatedPasefMsmsScans table TRUE | Max. peptide mass [Da] 4600  Min. peptide length for unspecific search 8  Max. peptide length for unspecific search 25  Razor protein FDR True  Disable MD5 False  Max mods in site table 3  Match unidentified features True  MS/MS tol. (FTMS) 20 ppm  Top MS/MS peaks per Da interval. (FTMS) 12  Da interval. (FTMS) 100  MS/MS deisotoping (FTMS) True  MS/MS deisotoping tolerance (FTMS) 7  MS/MS deisotoping tolerance unit (FTMS) ppm  MS/MS higher charges (FTMS) True  MS/MS water loss (FTMS) True  MS/MS ammonia loss (FTMS) True  MS/MS dependent losses (FTMS) True  MS/MS recalibration (FTMS) False  MS/MS tol. (ITMS) 0.5 Da  Top MS/MS peaks per Da interval. (ITMS) 8  Da interval. (ITMS) 100  MS/MS deisotoping (ITMS) False  MS/MS deisotoping tolerance (ITMS) 0.15  MS/MS deisotoping tolerance unit (ITMS) Da  MS/MS higher charges (ITMS) True  MS/MS water loss (ITMS) True  MS/MS ammonia loss (ITMS) True  MS/MS dependent losses (ITMS) True  MS/MS recalibration (ITMS) False  MS/MS tol. (TOF) 40 ppm  Top MS/MS peaks per Da interval. (TOF) 10  Da interval. (TOF) 100  MS/MS deisotoping (TOF) True  MS/MS deisotoping tolerance (TOF) 0.01  MS/MS deisotoping tolerance unit (TOF) Da  MS/MS higher charges (TOF) True  MS/MS water loss (TOF) True  MS/MS ammonia loss (TOF) True  MS/MS dependent losses (TOF) True  MS/MS recalibration (TOF) False  MS/MS tol. (Unknown) 0.5 Da  Top MS/MS peaks per Da interval. (Unknown) 8  Da interval. (Unknown) 100  MS/MS deisotoping (Unknown) False  MS/MS deisotoping tolerance (Unknown) 0.15  MS/MS deisotoping tolerance unit (Unknown) Da  MS/MS higher charges (Unknown) True  MS/MS water loss (Unknown) True  MS/MS ammonia loss (Unknown) True  MS/MS dependent losses (Unknown) True  MS/MS recalibration (Unknown) False  Site tables Oxidation (M)Sites.txt |

**Supplement Table 1** – *MaxQuant Settings for proteomic analysis – An overview of the MaxQuant settings used for quantitative analysis of acquired mass spectrometry data sets.*
